# Supplementary material for: Role and mechanism of NCAPD3 in promoting malignant behaviors in gastric cancer
Source: Front Pharmacol. 2024 Apr 22;15:1341039. doi: 10.3389/fphar.2024.1341039 (PMC11070777; doi:10.3389/fphar.2024.1341039)
Supplement: Supplementary file 11 [file DataSheet2.ZIP › GSEA/Transcipt factor/my_analysis.Gsea.1599461814734/LMO2COM_02.html]

Details for gene set LMO2COM\_02[GSEA]

|  || Dataset | filtered\_dataset.sample\_info.cls#WT\_versus\_NCAPD3\_MUT |
| Phenotype | sample\_info.cls#WT\_versus\_NCAPD3\_MUT |
| Upregulated in class | NCAPD3\_MUT |
| GeneSet | LMO2COM\_02 |
| Enrichment Score (ES) | -0.50501096 |
| Normalized Enrichment Score (NES) | -2.1445007 |
| Nominal p-value | 0.0 |
| FDR q-value | 0.04111863 |
| FWER p-Value | 0.252 |
Table: GSEA Results Summary

  

Fig 1: Enrichment plot: LMO2COM\_02      
 Profile of the Running ES Score & Positions of GeneSet Members on the Rank Ordered List

  

| SYMBOL | TITLE | RANK IN GENE LIST | RANK METRIC SCORE | RUNNING ES | CORE ENRICHMENT || 1 | 54462 | CCSER2 | 287 | 0.603 | -0.1420 | No |
| 2 | 1601 | DAB2 | 327 | 0.577 | -0.1089 | No |
| 3 | 805 | CALM2 | 423 | 0.518 | -0.1222 | No |
| 4 | 3164 | NR4A1 | 912 | -0.341 | -0.4361 | No |
| 5 | 9586 | CREB5 | 1009 | -0.409 | -0.4616 | Yes |
| 6 | 10019 | SH2B3 | 1044 | -0.440 | -0.4394 | Yes |
| 7 | 80315 | CPEB4 | 1053 | -0.448 | -0.3977 | Yes |
| 8 | 1191 | CLU | 1056 | -0.450 | -0.3514 | Yes |
| 9 | 339122 | RAB43 | 1083 | -0.462 | -0.3210 | Yes |
| 10 | 604 | BCL6 | 1136 | -0.504 | -0.3049 | Yes |
| 11 | 146057 | TTBK2 | 1148 | -0.513 | -0.2584 | Yes |
| 12 | 639 | PRDM1 | 1159 | -0.523 | -0.2101 | Yes |
| 13 | 831 | CAST | 1160 | -0.524 | -0.1546 | Yes |
| 14 | 2355 | FOSL2 | 1262 | -0.648 | -0.1584 | Yes |
| 15 | 861 | RUNX1 | 1293 | -0.687 | -0.1071 | Yes |
| 16 | 4622 | MYH4 | 1355 | -0.811 | -0.0650 | Yes |
| 17 | 8867 | SYNJ1 | 1387 | -0.979 | 0.0165 | Yes |
Table: GSEA details [plain text format]

  

Fig 2: LMO2COM\_02      
 Blue-Pink O' Gram in the Space of the Analyzed GeneSet

  

Fig 3: LMO2COM\_02: Random ES distribution      
 Gene set null distribution of ES for **LMO2COM\_02**
